# Pumilio2-deficient mice show a predisposition for epilepsy

**DOI:** 10.1242/dmm.029678

**Published:** 2017-11-01

**Authors:** Philipp Follwaczny, Rico Schieweck, Therese Riedemann, Antonia Demleitner, Tobias Straub, Anna H. Klemm, Martin Bilban, Bernd Sutor, Bastian Popper, Michael A. Kiebler

**Affiliations:** 1 Biomedical Center (BMC), Department for Cell Biology, Faculty of Medicine, LMU, Munich, 82152 Planegg-Martinsried, Germany; 2 Biomedical Center (BMC), Department of Physiological Genomics, Ludwig-Maximilians-University, Munich, 82152 Planegg-Martinsried, Germany; 3 Biomedical Center (BMC), Core Facility Bioinformatics, Ludwig-Maximilians-University, Munich, 82152 Planegg-Martinsried, Germany; 4 Biomedical Center (BMC), Core Facility Bioimaging, Ludwig-Maximilians-University, Munich, 82152 Planegg-Martinsried, Germany; 5 Walter Brendel Centre of Experimental Medicine, Ludwig-Maximilians-University, 81377 Munich, Germany; 6 Department of Laboratory Medicine and Core Facility Genomics, Medical University of Vienna, 1090 Vienna, Austria; 7 Biomedical Center (BMC), Core Facility Animal Models, Ludwig-Maximilians-University, Munich, 82152 Planegg-Martinsried, Germany

**Keywords:** RNA-binding protein, Pumilio2, PUM2, Epilepsy, Epileptogenesis, Risk factor

## Abstract

Epilepsy is a neurological disease that is caused by abnormal hypersynchronous activities of neuronal ensembles leading to recurrent and spontaneous seizures in human patients. Enhanced neuronal excitability and a high level of synchrony between neurons seem to trigger these spontaneous seizures. The molecular mechanisms, however, regarding the development of neuronal hyperexcitability and maintenance of epilepsy are still poorly understood. Here, we show that pumilio RNA-binding family member 2 (Pumilio2; Pum2) plays a role in the regulation of excitability in hippocampal neurons of weaned and 5-month-old male mice. Almost complete deficiency of Pum2 in adult *Pum2* gene-trap mice (Pum2 GT) causes misregulation of genes involved in neuronal excitability control. Interestingly, this finding is accompanied by the development of spontaneous epileptic seizures in Pum2 GT mice. Furthermore, we detect an age-dependent increase in *Scn1a* (Na_v_1.1) and *Scn8a* (Na_v_1.6) mRNA levels together with a decrease in *Scn2a* (Na_v_1.2) transcript levels in weaned Pum2 GT that is absent in older mice. Moreover, field recordings of CA1 pyramidal neurons show a tendency towards a reduced paired-pulse inhibition after stimulation of the Schaffer-collateral-commissural pathway in Pum2 GT mice, indicating a predisposition to the development of spontaneous seizures at later stages. With the onset of spontaneous seizures at the age of 5 months, we detect increased protein levels of Na_v_1.1 and Na_v_1.2 as well as decreased protein levels of Na_v_1.6 in those mice. In addition, GABA receptor subunit alpha-2 (*Gabra2*) mRNA levels are increased in weaned and adult mice. Furthermore, we observe an enhanced GABRA2 protein level in the dendritic field of the CA1 subregion in the Pum2 GT hippocampus. We conclude that altered expression levels of known epileptic risk factors such as Na_v_1.1, Na_v_1.2, Na_v_1.6 and GABRA2 result in enhanced seizure susceptibility and manifestation of epilepsy in the hippocampus. Thus, our results argue for a role of Pum2 in epileptogenesis and the maintenance of epilepsy.

## Abstract (precis)

**Summary:** Epileptogenic risk factors are misregulated in Pumilio2-deficient mice, determining a predisposition to develop seizures. This article has an associated First Person interview with the first author of the paper as part of the supplementary information.

## INTRODUCTION

Epilepsy is one of the most common neurological diseases in humans. It is characterized by the occurrence of spontaneous seizures (Pernice et al., 2016). These seizures can be caused by hyperexcitability of neurons as well as hypersynchronous network activity. Great effort has been made to identify possible risk factors responsible for epileptogenesis (Bertram, 2003). Among others, voltage-gated sodium and potassium channels as well as the γ-aminobutyric acid receptor A (GABA_A_)-receptor family have particularly been linked to epilepsy in animal models and human patients (Staley, 2015). It remains elusive, however, how those proteins act together during development and maintenance of epilepsy in adulthood.

Research in the last decades unraveled that RNA-binding proteins (RBPs) control the expression of their target RNAs (Jung et al., 2014). Thereby, they provide another regulation level to guide remote protein expression. One of the best characterized RBPs is the fragile-X mental retardation protein (FMRP). Loss of FMRP causes fragile-X syndrome (Pieretti et al., 1991), a disease that is hallmarked by mental retardation and the occurrence of seizures (Darnell and Klann, 2013). Therefore, it has been suggested that RBPs play an important role in the development and maintenance of healthy homeostasis in the brain. The RBP pumilio RNA-binding family member 2 (Pumilio2; Pum2) is a posttranscriptional regulator whose function is conserved from yeast to human (Quenault et al., 2011). Pum2 binds an eight-nucleotide consensus sequence in the 3′-untranslated region (3′-UTR) of its target mRNAs (White et al., 2001). Thereby, it regulates the expression of the encoded protein. In addition, Pum2 controls the expression of the voltage-gated sodium channel (Na_v_) Na_v_1.6 and dendrite morphogenesis of dissociated hippocampal neurons (Driscoll et al., 2013; Vessey et al., 2010), indicating a role in the regulation of neuronal excitability. Furthermore, Pum2 was reported to be downregulated in two epilepsy models in *Drosophila* (Lin et al., 2017). Moreover, knockdown of Pum2 in mice has been shown to cause spontaneous epileptic seizures (Siemen et al., 2011). In the study presented here, we investigated the molecular mechanisms of Pum2-loss-induced spontaneous epileptic seizures and present the first evidence of how Pum2 deficiency might cause late-onset epilepsy in *Pum2* gene-trap (Pum2 GT) mice.

Here, we took advantage of a previously published Pum2 GT mouse model that shows Pum2 deficiency (Siemen et al., 2011). Male mice that are largely deficient of Pum2 develop spontaneous epileptic seizures in adulthood, mainly at the age of 5 months. In order to investigate the underlying mechanism of the development of spontaneous seizures, we analyzed mRNA levels of ion channels, ion transporters and receptors that guide neuronal excitability, and found these to be dysregulated in the absence of Pum2. In detail, we observed age-dependent alterations of mRNA and protein levels for *Scn1a* (Na_v_1.1) and *Scn8a* (Na_v_1.6) in the brain of weaned and 5-month-old mice. Strikingly, we detected a twofold upregulation of γ-aminobutyric acid receptor A (GABA) subunit α2 (*Gabra2*) mRNA for both ages tested. Strikingly, electrophysiological recordings of the Schaffer-collateral-commissural (SCC) pathway revealed reduced paired-pulse inhibition. Furthermore, we observed enhanced dendritic localization of the GABRA2 subunit in hippocampal CA1 neurons. Together, these findings suggest a role of Pum2 in the development and maintenance of epilepsy in adulthood that is, *inter alia*, mediated by altered neuronal inhibition.

## RESULTS

### Brain-wide Pum2 knockdown leads to misregulation of genes associated with epilepsy

**Fig. 1.**
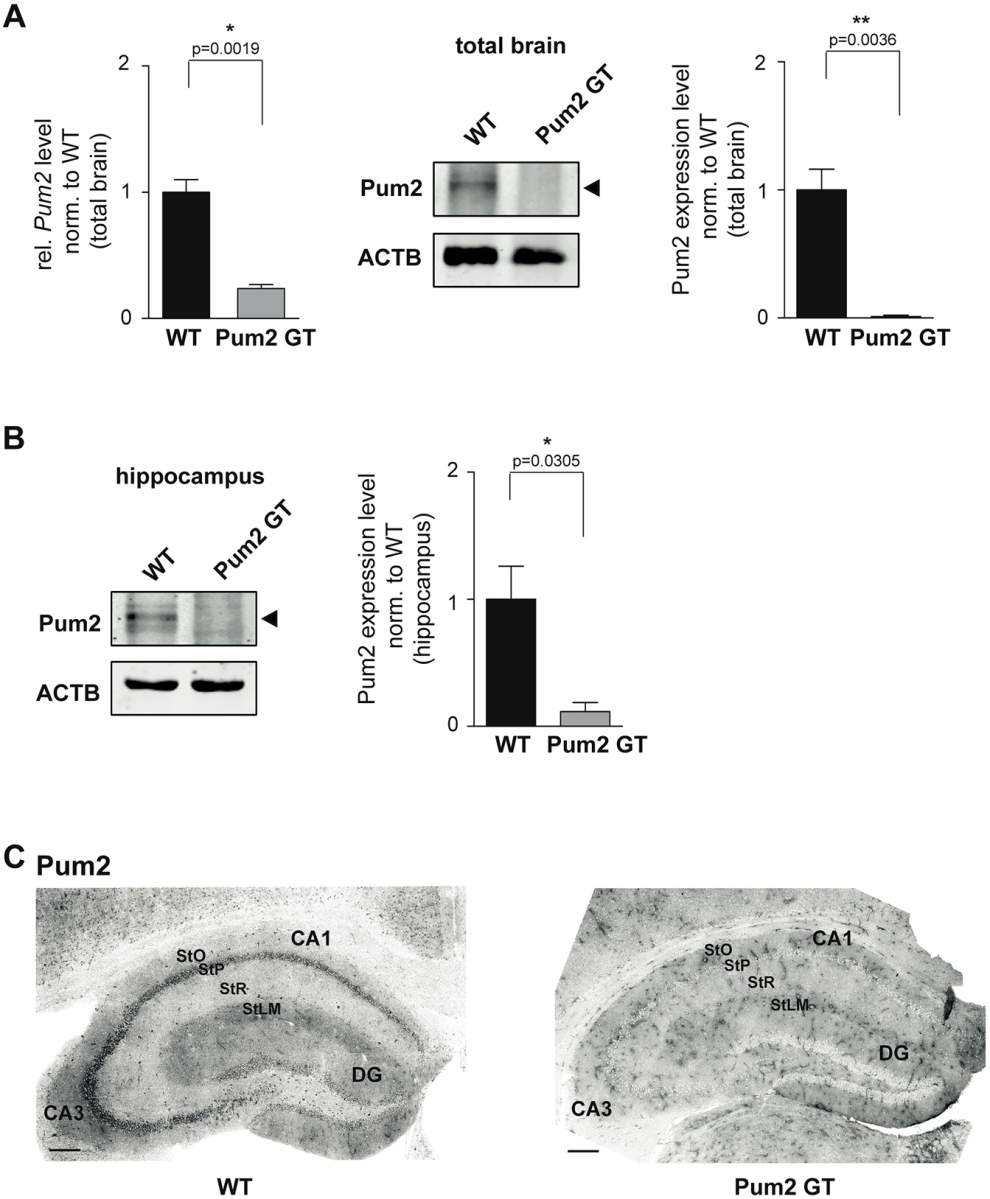
**Gene-trap (GT) vector insertion reduces Pum2 expression level in the hippocampus.** (A) qRT-PCR of *Pum2* mRNA levels (left) and western blot analysis of Pum2 protein levels (middle: representative western blot; right: quantification) of WT and Pum2 GT mouse brain lysates. β-actin (ACTB) was used as loading control (*n*=3 animals/group). Significance was determined using unpaired *t*-test. *P<0.05. (B) Representative western blot and quantification of Pum2 protein levels in homogenates obtained from WT and Pum2 GT hippocampi. β-actin (ACTB) was used as loading control (*n*=3 animals/group). Significance was determined using unpaired *t*-test. *P<0.05. (C) Immunohistological stainings of WT and Pum2 GT hippocampus (coronal sections). Scale bars: 200 µm. StO, stratum oriens; StP, stratum pyramidale; StR, stratum radiatum; StLM, stratum lacunosum-moleculare; DG, dentate gyrus.

To investigate the effect of Pum2 knockdown on epilepsy risk-factor expression, we took advantage of an existing Pum2 GT mouse exhibiting reduced Pum2 expression levels (Siemen et al., 2011; Xu et al., 2007). Quantitative reverse-transcription PCR (qRT-PCR) of total RNA from brains revealed an 80% reduction of *Pum2* mRNA (Fig. 1A, left) and more than 90% for the corresponding protein (Fig. 1A, middle, quantification right). Similar results were obtained for Pum2 protein levels in the hippocampus (Fig. 1B, quantification right). Immunohistochemistry of coronal hippocampal sections showed a prominent Pum2 signal in the pyramidal cell layers (CA3-CA1) and less intense in the granular cell layer [dentate gyrus (DG)] that was absent in the hippocampus of Pum2 GT mice (Fig. 1C).

**Fig. 2.**
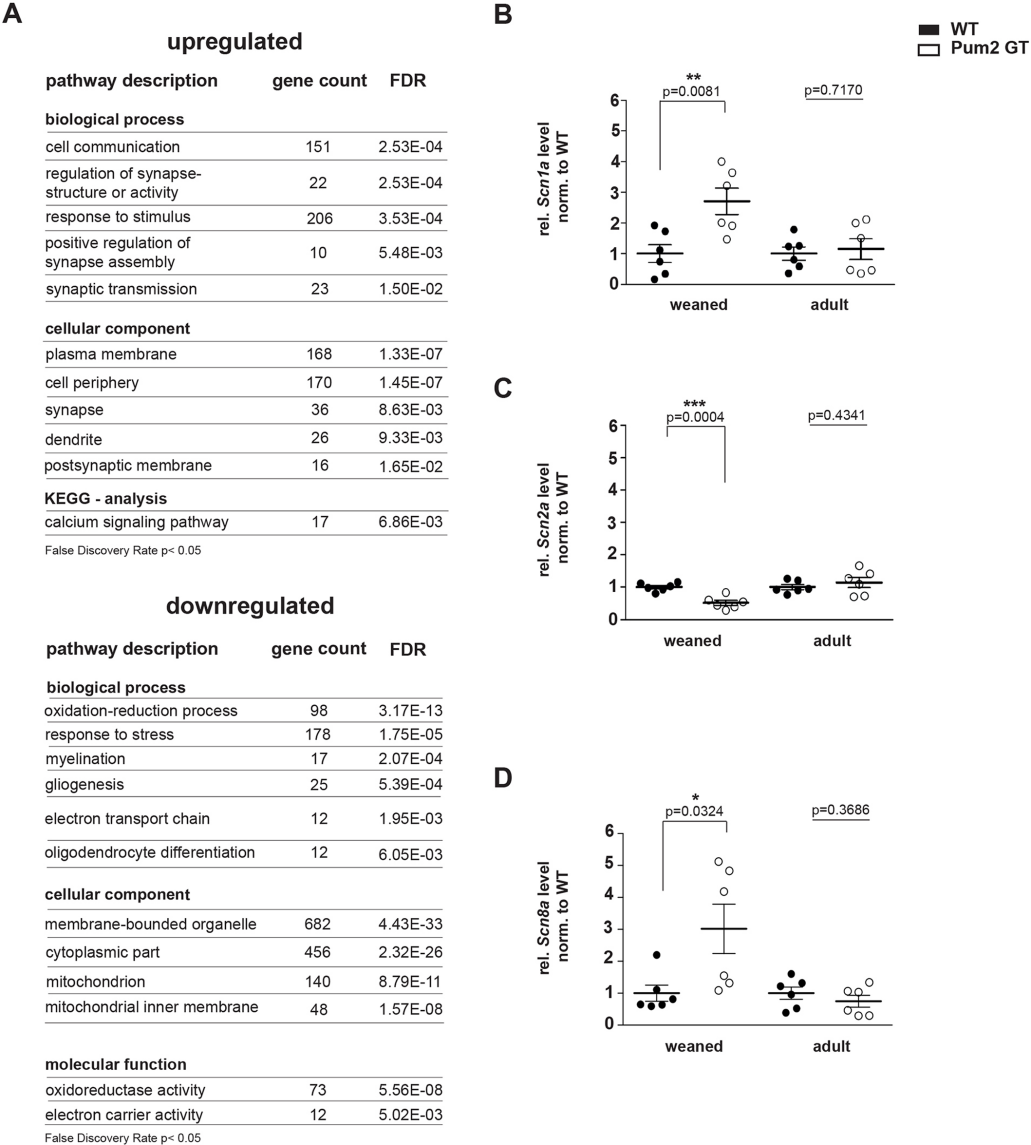
**Epileptogenic factors are misregulated in Pum2 GT mice.** (A) Gene ontology (GO) classification of mRNAs identified by microarray analysis that are upregulated (top) and downregulated (bottom) in Pum2 GT compared to WT mice (*n*=3 animals/group). FDR, false discovery rate. (B-D) qRT-PCR mRNA expression analysis for *Scn1a* (B), *Scn2a* (C) and *Scn8a* (D) coding for Na_v_1.1, Na_v_1.2 and Na_v_1.6, respectively, in total brain lysates obtained from weaned (3-week-old) and 5-month-old Pum2 GT and WT mice (*n*=6 animals for all groups). Significance was determined using unpaired *t*-test. *P<0.05, **P<0.01, ***P<0.001.

In previous studies, it has been shown that *Pum2* mRNA targets *Scn1a* and *Scn8a* mRNAs (Driscoll et al., 2013; Vessey et al., 2010). In addition, bioinformatic analysis of known epileptic risk factors revealed a possible Pum2-binding site in the 3′-UTR of *Scn1a* and *Scn8a* mRNAs. These results suggest that Pum2 is involved in the regulation of voltage-gated sodium-channel expression and thereby might control neuronal excitability in mice. To get further insight into the expression levels of target mRNAs in the absence of Pum2, we performed a transcriptome-wide microarray analysis in Pum2 GT and wild-type (WT) brains at the age of 5 months, the time of onset of spontaneous epileptic seizures in Pum2 GT mice. Strikingly, we found mRNAs coding for proteins involved in cell communication and synaptic transmission to be upregulated (Fig. 2A). Among others, our microarray analysis revealed an altered expression level of transporters for sodium, potassium and calcium ions (Table S1). Interestingly, we also observed the translational repressors *Nanos2* and *Nanos3* to be upregulated and mRNAs encoding for components of the eukaryotic initiation factor 3 complex (eIF3) to be downregulated (Table S1). Strikingly, *Gabra2*, which has been linked to epilepsy in humans (Loddenkemper et al., 2014), was upregulated twofold. For known Pum2 targets such as *Scn1a* and *Scn8a*, we did not detect changes at the mRNA level in 5-month-old brains. The transcriptome data described above served as a starting point to further test expression levels of these well-known epilepsy genes. Therefore, we performed qRT-PCR for *Scn1a* and *Scn8a*, coding for the voltage-gated sodium channels Na_v_1.1 and 1.6, as well as for the epilepsy gene *Scn2a*, coding for Na_v_1.2, in brain lysates from weaned and 5-month-old mice. Pum2 GT mice show spontaneous epileptic seizures at the age of 5 months (Siemen et al., 2011). We chose this age for mRNA quantification to investigate the onset of epileptic seizures. In addition, we analyzed mRNA levels of the above-mentioned targets in weaned animals [postnatal day 21 (P21)] in order to address the effect of Pum2 deficiency on the development and establishment of neuronal activity during late brain development (Fig. 2B-D). Interestingly, mRNAs coding for Na_v_1.1 and Na_v_1.6 showed a strong upregulation in weaned Pum2 GT mice compared to WT. We did not observe this effect in 5-month-old animals (Fig. 2B,D). In addition, *Scn2a* (Na_v_1.2) mRNA levels were reduced in weaned Pum2 GT animals and returned to control values at the age of 5 months (Fig. 2C). Thus, our results suggest that *Scn1a*, *Scn2a* and *Scn8a* expression is dynamically regulated during postnatal development in response to Pum2 knockdown.

### Increased paired-pulse ratios in CA1 pyramidal cells of Pum2 GT mice after SCC pathway stimulation

**Fig. 3.**
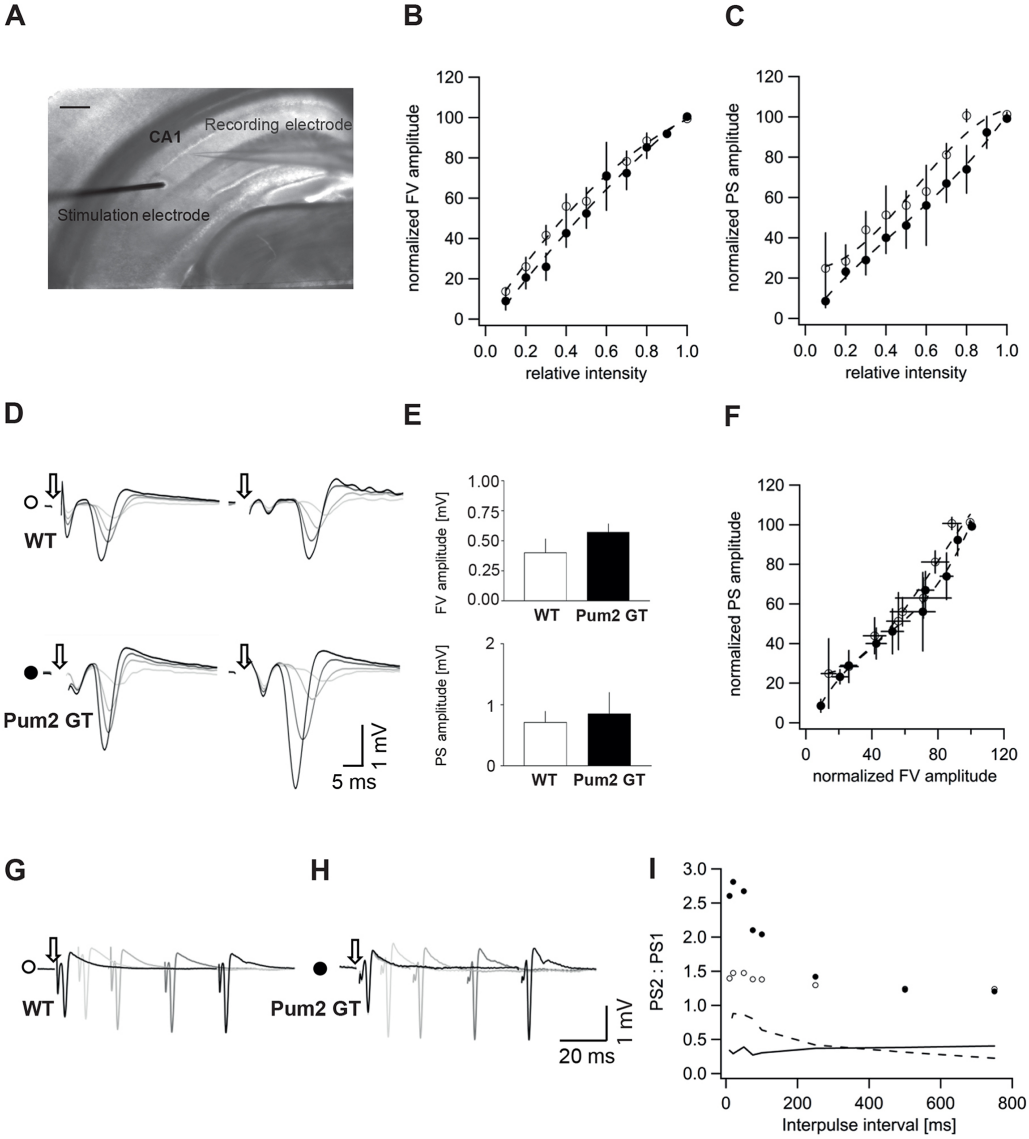
**Field recordings of acute hippocampal slices after Schaffer-collateral-commissural (SCC) pathway stimulation show reduced paired-pulse inhibition in Pum2 GT mice.** (A) Representative bright-field image of the recording setup: a monopolar or bipolar stimulation electrode was placed onto the SCC pathway and the corresponding population spike was recorded from the pyramidal layer of the CA1 region of the hippocampus. Scale bar: 500 µm. (B) Input-output analysis of evoked fiber volley (FV) responses in WT (white circles) and Pum2 GT (black circles) mice (*n*=5 animals/group), represented as the normalized FV amplitude (given as percentage of the maximal amplitude) as a function of the relative stimulation intensity (as percentage of the maximal current intensity). Data are means±s.e.m. (C) Input-output analysis of evoked population spike (PS) responses in WT (white circles) and Pum2 GT (black circles) mice. The normalized PS amplitude is plotted as a function of the relative current intensity (*n*=5 animals/group). (D) Representative single PS traces after stimulation with the following relative current intensities: 0.3 (light gray), 0.6 (medium gray), 0.8 (dark gray) and 1.0 (black) units. White circle: WT mouse; black circle: Pum2 GT mouse. Arrows indicate the stimulation onset; the stimulation artefact was removed from the single traces. (E) Overall FV (top) or PS (bottom) amplitudes in WT and Pum2 GT slides (*n*=5 animals/group). Data are means±s.e.m. (F) Normalized PS amplitude is plotted as a function of the normalized presynaptic FV amplitude (*n*=5 animals/group). Data are means±s.e.m. (G,H) Single traces of PS in WT mice (G) and Pum2 GT mice (H) after paired-pulse stimulations with different interpulse intervals (IPIs) are overlaid. Black trace: IPI 100 ms; dark gray trace: IPI 75 ms; medium gray trace: IPI 20 ms; light gray trace: IPI 10 ms. Arrows indicate the onset of stimulation; the stimulation artefact was truncated. (I) Ratio of the amplitude of the second PS (PS2) compared to the first one (PS1) as a function of the IPI. The black solid line indicates the coefficient of variance (CV) of recordings from WT mice; the dashed line indicates the CV of PS2:PS1 ratios in Pum2 GT mice. WT, white circles; Pum2 GT, black circles.

In order to get further insight into the development of spontaneous seizures in adult (P70-P84; no spontaneous epileptic seizures were yet observed at this age) Pum2 GT mice, we performed field recordings in acute hippocampal slices. Evoked population spikes in CA1 pyramidal neurons were recorded after SCC pathway stimulation. After correct positioning of the stimulation and recording electrode (Fig. 3A), we performed an input-output analysis and analyzed the normalized amplitudes of the presynaptic fiber volley (FV) as well as of the population spike (PS) as a function of the stimulation intensity (Fig. 3B,C). The stimulus-response relation of PS amplitudes in control and Pum2 GT mice were similar, indicating no alterations in the overall network excitability. This finding was further supported by the fact that we failed to detect significant differences in the magnitude of the FV or PS responses (Fig. 3D,E). However, in three out of five slices from Pum2 GT mice, we did detect a higher probability for multiple population spikes in response to afferent stimulation. Moreover, excitability was analyzed by plotting the PS amplitude as a function of the FV amplitude, and we detected no differences in WT and Pum2 GT mice (Fig. 3F). Next, in order to measure the ability of hippocampal interneurons to inhibit subsequent population responses, we performed paired-pulse stimulations at different interpulse intervals (IPIs), ranging from 750 ms to 20 ms IPI, at a stimulation intensity of around 60% (Fig. 3G,H). Paired-pulse ratios (PPRs) were plotted as a function of the IPI and we found a higher tendency in Pum2 GT mice for decreased paired-pulse inhibition compared to control mice, suggestive of reduced network inhibition (Fig. 3I). We conclude that this reduced network inhibition might be a cause for the development of spontaneous epileptic seizures.

### Altered expression of sodium channels with the onset of spontaneous seizures

**Fig. 4.**
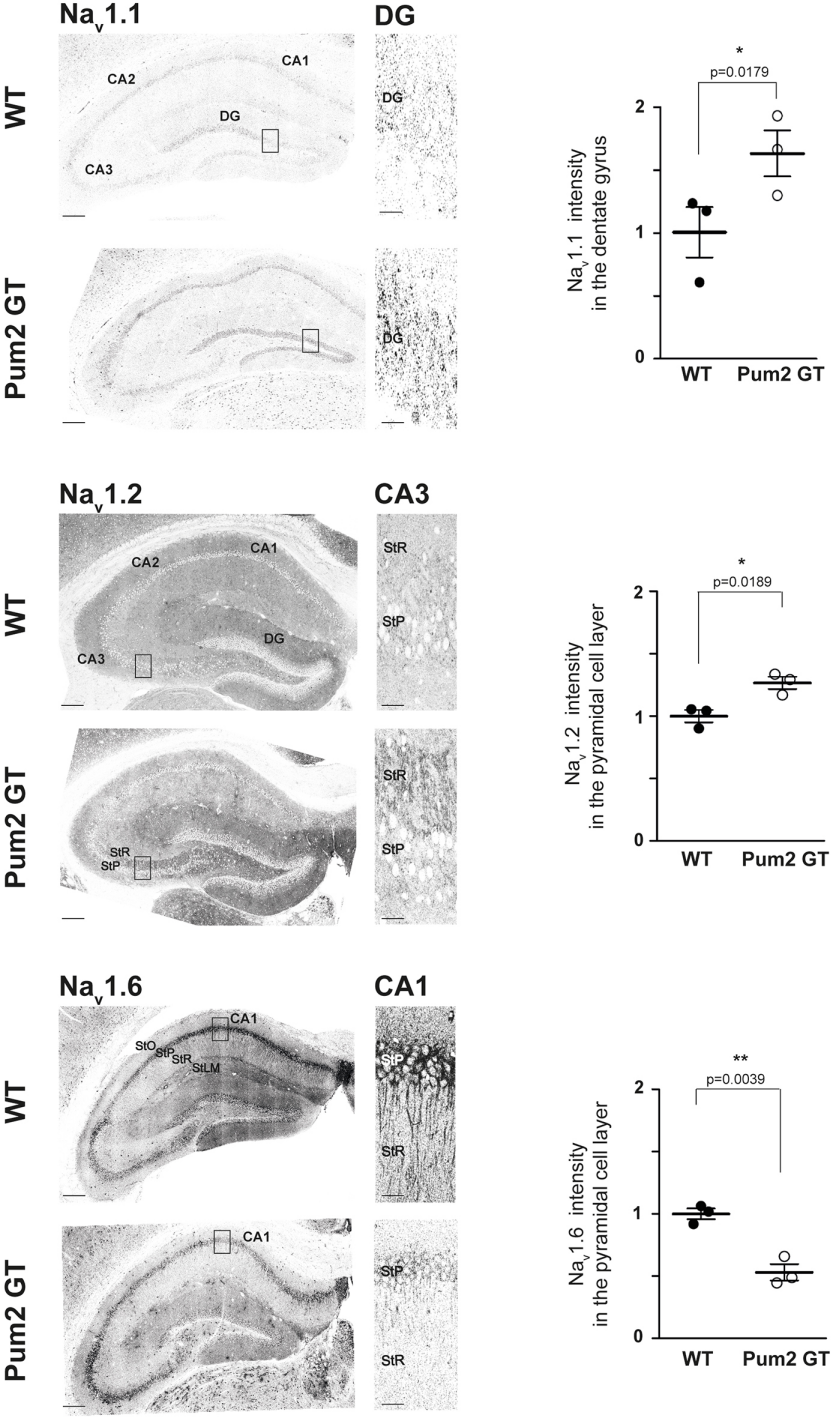
**Pum2 knockdown affects expression of Na_v_ channels in different hippocampal areas.** Immunohistological stainings for Na_v_1.1, Na_v_1.2 and Na_v_1.6 of the hippocampus of 5-month-old WT and Pum2 GT mice. Boxed areas indicate the magnified field that is shown. Quantification is shown for dentate gyrus (DG; Na_v_1.1) and pyramidal cell layer (Na_v_1.2, Na_v_1.6) (*n*=3 animals for all groups). Scale bars: 200 µm; insets: 20 µm. Significance was determined using unpaired *t*-test. *P<0.05.

Pum2 is highly expressed in the hippocampus (Allen Brain Atlas: www.brain-map.org/). The occurrence of epileptic seizures and, eventually, epilepsy is caused by disturbed excitability mediated, *inter alia*, by voltage-gated sodium channels. Within those, Na_v_1.1, Na_v_1.2 and Na_v_1.6 have been linked to epilepsy in human patients (Oliva et al., 2012). To test for protein expression levels of Na_v_1.1, Na_v_1.2 and Na_v_1.6, we performed immunohistochemistry on coronal brain slices of the dorsal hippocampus (Fig. 4). All Na_v_ channels tested showed a staining pattern that followed the *in situ* hybridization results of the Allen Brain Atlas. In addition, we observed a clear dendritic localization for Na_v_1.6 in the CA1 subregion in the WT hippocampus that is reduced in Pum2 GT mice (Fig. S2A). Strikingly, fluorescent signal quantifications showed significantly altered protein expressions of Na_v_ channels in the hippocampus of 5-month-old Pum2 GT mice. Whereas protein levels of Na_v_1.1 and Na_v_1.2 were increased, Na_v_1.6 protein showed a reduced staining intensity. However, we did not detect statistically significant differences in staining intensity for Na_v_1.1, Na_v_1.2 and Na_v_1.6 in the hippocampus of weaned mice (WT versus Pum2 GT) (Fig. S1).

### Loss of Pum2 impacts GABRA2 expression levels and localization in CA1 pyramidal neurons

**Fig. 5.**
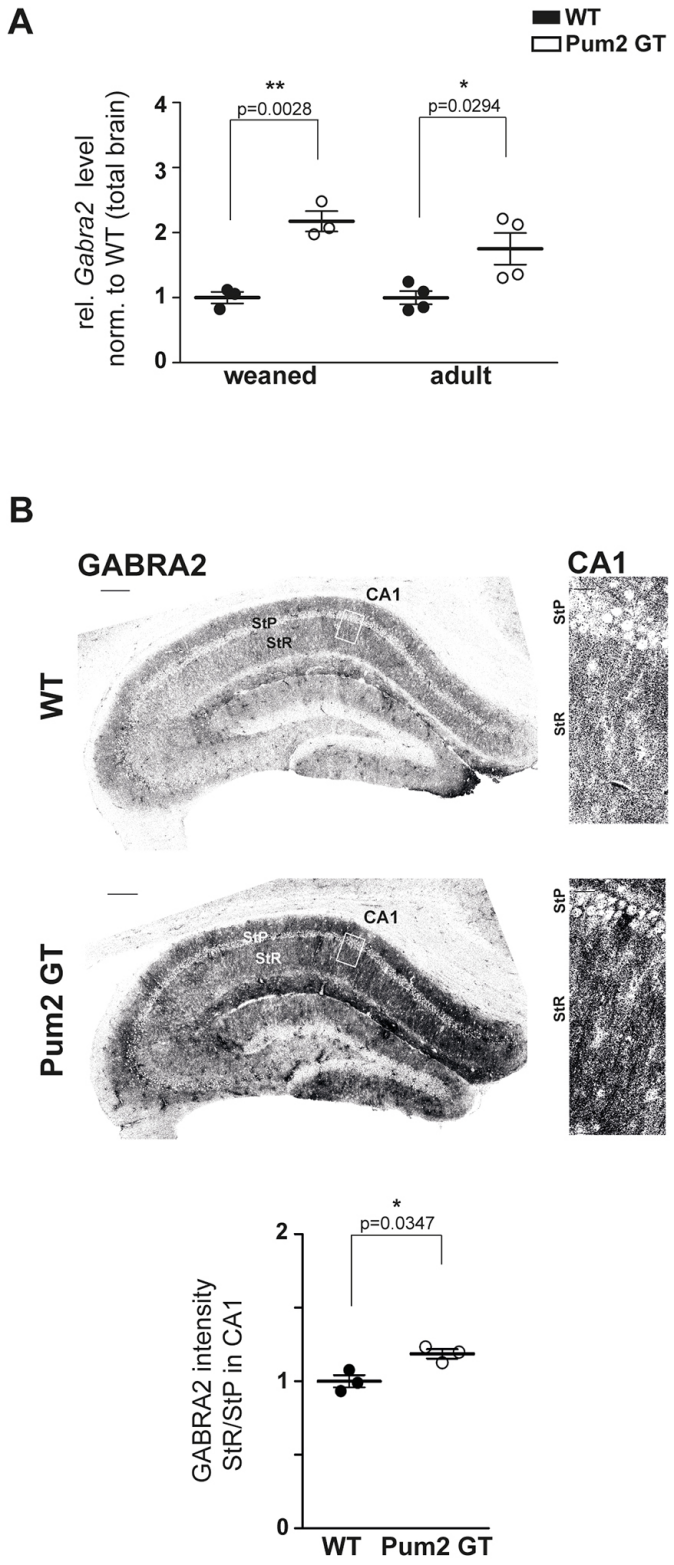
**GABRA2 shows increased mRNA levels and enhanced dendritic protein localization in the hippocampus of adult Pum2 GT mice.** (A) *Gabra2* mRNA levels are quantified by qRT-PCR in 3-month-old (weaned) and 5-month-old WT and Pum2 GT brains (*n*≥3 animals/group). (B) Representative staining of GABRA2 in the hippocampus of 5-month-old WT and Pum2 GT animals, and quantification of GABRA2 protein expression in the stratum radiatum (StR) compared to stratum pyramidale (StP) in the CA1 area of WT and Pum2 GT mice. Scale bars: 200 µm; insets: 20 µm. Significance was determined using unpaired *t*-test. *P<0.05.

GABA_A_ receptors are chloride ion channels activated by the neurotransmitter GABA that have been linked to epilepsy (Loddenkemper et al., 2014). Our transcriptome analysis revealed a twofold upregulation of *Gabra2* levels in adult Pum2 GT mice that we confirmed by qRT-PCR in weaned and 5-month-old mice (Fig. 5A). This effect is specific for *Gabra2* and not a general effect of GABA receptor expression because γ-aminobutyric acid receptor B subunit 2 (*Gabbr2*), a member of the GABA_B_-receptor family, remained unaffected (Fig. S2E). To test for alterations in protein levels, we performed immunohistochemistry on coronal slices of the dorsal hippocampus with antibodies specific for GABRA2 (Quadrato et al., 2014). Interestingly, we detected a significantly higher staining intensity in the dendritic field [stratum radiatum (StR)] of CA1 neurons compared to the pyramidal cell layer [stratum pyramidale (StP)] in 5-month-old Pum2 GT mice (Fig. 5B; Fig. S2B,C). Importantly, the expression levels of GABRA2 in pyramidal cells of the CA3-CA1 subregions and in granule cells in the DG as well as in the corresponding dendritic fields remained unaffected.

In summary, our expression analysis show that knockdown of Pum2 affects the expression of *Gabra2* age-independently, in contrast to the tested Na_v_ channels. Furthermore, Pum2 GT mice show reduced network inhibition. Thus, our results suggest that neuronal inhibition is mostly affected in Pum2 GT mice.

## DISCUSSION

In this study, we investigated the age-dependent expression of known epileptogenic genes in Pum2-deficient mouse brains. In order to investigate the effect of Pum2 knockdown on mRNA levels, we performed a transcriptome-wide microarray analysis. Strikingly, gene ontology (GO) analysis revealed significantly enriched categories for genes involved in cell communication and synaptic transmission – two processes known to be affected in epilepsy models (Staley, 2015). Furthermore, Pum2 deficiency resulted in altered expression levels for a subset of genes coding for proteins involved in neuronal excitability. Among those genes, using microarray and qRT-PCR experiments, *Gabra2* levels were shown to be upregulated by 100% in weaned and adult Pum2 GT brains. Interestingly, we also detected altered *Scn1a*, *Scn2a* and *Scn8a* expression levels in weaned but not in 5-month-old Pum2 GT mice. According to binding-site screening in the 3′-UTR, all four candidate genes have a Pum2 consensus sequence in their 3′-UTR. It has been shown that Pum2 recruits the deadenylase complex CCR4-NOT (Van Etten et al., 2012), which has been linked to RNA decay (Collart, 2016). Therefore, it is tempting to speculate that the upregulation in expression levels for *Scn1a*, *Scn8a* and *Gabra2* might be caused by increased mRNA stability in the absence of Pum2.

Additionally, it has been shown that Pum2 regulates the translation of Na_v_1.6 in dissociated hippocampal neurons (Driscoll et al., 2013). Therefore, we tested protein expression levels for Na_v_1.1, Na_v_1.2 and Na_v_1.6 in the hippocampus. Interestingly, we observed higher Na_v_1.1 and Na_v_1.2 levels in both the DG and pyramidal cell layer, respectively, but reduced Na_v_1.6 levels in the pyramidal cell layer in 5-month-old Pum2 GT brains. In weaned mice, protein levels are not significantly altered.

We suggest that there are at least two possible explanations for the differences in mRNA and protein levels for *Scn1a*, *Scn2a* and *Scn8a*: (i) protein levels for the sodium channels are significantly changed in weaned mice in extrahippocampal regions such as forebrain or (ii) other translation repressors, such as *Nanos*, inhibit the translation of these sodium channels as a compensatory effect. For 5-month-old Pum2 GT mice, unaffected RNA levels but altered protein levels argue for translational regulation.

Next, in order to test for a functional impact of Pum2 knockdown on neuronal activity, we recorded evoked population spikes in CA1 pyramidal neurons after SCC pathway stimulation. Importantly, a previous study has shown that Pum2 GT animals show abnormal discharging in EEG recordings (Siemen et al., 2011). Our results suggest that Pum2 has no impact on overall neuronal activity. CA1 pyramidal neurons do not seem to exhibit a higher excitability level in Pum2 GT mice compared with WT. This is in line with our observation that weaned mice show no differences in the protein expression of Na_v_1.1, Na_v_1.2 and Na_v_1.6. We found that Pum2 knockdown resulted in increased Na_v_1.1 and Na_v_1.2 levels in 5-month-old mice, a finding that could possibly be accompanied by a higher excitability of principal cells after afferent stimulation. A likely explanation(s) for the fact that we did not observe increased excitability in Pum2 GT mice might be that: (i) principal cells as well as interneurons show a similar increase in Na_v_ protein levels, therefore maintaining the overall excitation to inhibition ratio; (ii) Na_v_ protein levels of β-subunits that regulate the gating behavior of their associated α-subunits are reduced/dysfunctional; or (iii) the analyzed young adult mice had not yet developed spontaneous seizures and, therefore, a clear phenotype is not yet detectable. We suggest that the generation of spontaneous seizures in 5-month-old Pum2 GT mice is likely due to reduced network inhibition and less likely due to increased neuronal excitability. This hypothesis is further supported by the finding that we detected a tendency towards reduced inhibition after paired-pulse stimulation in Pum2 GT mice compared to controls. In agreement with this interpretation, it is known that dysfunctional or loss of GABAergic inhibition can cause paroxysmal activity and a loss of paired-pulse inhibition (Kapur et al., 1989a,b; Sloviter and Brisman, 1995).

Loss of paired-pulse inhibition, indicative of reduced GABAergic inhibition, points towards a reduction of GABRA2 levels. However, we detected an upregulation of *Gabra2* levels in Pum2 GT brains. It remains to be investigated whether these increased GABRA2 levels are accompanied by increased expression of functional synaptic GABA_A_ receptors or whether this effect is specific for certain brain regions. Moreover, we detected an enhanced dendritic localization of GABRA2 in CA1 pyramidal neurons of Pum2 GT mice, possibly due to potentially higher neuronal input from CA3 neurons and/or the entorhinal cortex (Pettit and Augustine, 2009). Moreover, given the small volume of the dendritic compartment and thus a higher probability of a shift of its chloride equilibrium potential towards more positive values, increased dendritic localization of GABA_A_ receptors is able to actively contribute to action-potential induction in CA1 neurons of Pum2 GT mice (Jedlicka et al., 2011; Staley and Proctor, 1999). Moreover, depolarizing actions of GABA have been reported in neocortical pyramidal cells of adult mice (Gulledge and Stuart, 2003). Interestingly, a transition from dominant phasic GABAergic inhibition to dominant phasic GABAergic excitation has also been shown in a mouse model of epilepsy (Derchansky et al., 2008). Another possible explanation for reduced paired-pulse inhibition despite higher levels of dendritic GABA_A_ receptor expression could stem from findings showing that the function of chloride transporters that actively extrude chloride out of the cell can be impaired in epilepsy (Buchin et al., 2016; Conti et al., 2011; Doyon et al., 2016). It remains to be investigated whether chloride transporters, especially the K-Cl cotransporter KCC2, are affected by Pum2 knockdown.

It is generally believed that voltage-gated sodium channels and GABA_A_ receptors crucially contribute to the development and manifestation of epilepsy in human patients and animal models (Staley, 2015). In our study, we observed altered mRNA levels of *Scn1a*, *Scn2a* and *Scn8a* in weaned but not in adult mice. Based on our results, we conclude that brain-wide knockdown of Pum2 causes a predisposition in developing animals to develop epileptic seizures that might be mediated by altered mRNA levels of known epileptogenic factors. At this age, we did not observe differences on the corresponding protein level in the hippocampus. We speculate that this effect is, preferentially, due to increased translational repression. During epileptogenesis, mRNAs are released from repression, which then affects the protein levels in the hippocampus of adult, 5-month-old animals and leads to manifestation of spontaneous epileptic seizures (Siemen et al., 2011). In summary, the aim of the study presented here was to identify, in mice, epileptogenic risk factors during the development and maintenance of epilepsy that are known to increase the risk for epilepsy when misregulated. Together with the fact that Pum2 is downregulated in postmortem brains of patients who suffered from epileptic seizures (Wu et al., 2015), we conclude that Pum2 is a key regulator of epileptogenic risk factors.

## MATERIALS AND METHODS

### Mice

For all experiments, male mice homozygous for GT-vector insertion [B6.129P2-Pum2^GT(XE772)Byg^] in the *Pum2* locus (Pum2 GT) and WT control animals (genetic background for WT and Pum2 GT mice: C57Bl6/J) at the age of P21 (weaned) or 5 months (adult) were investigated. Pum2 GT mice were a gift from Dr Eugene Xu (Northwestern University, IL, USA). Mice were kept under specified pathogen-free conditions and housed in groups of two to five animals in individually ventilated cages and a 12 h/12 h light/dark cycle. Mice had free access to water and standard rodent chow. All experiments were approved by the authors' institutional committee on animal care and were performed according to the German Animal Protection Law, conforming to international guidelines on the ethical use of animals.

### Microarray analysis

RNA was isolated as described in qRT-PCR. Samples were processed according to the manufacturer's instructions (Affymetrix) and hybridized on a Mouse Gene 2.0 ST Array. Signal intensities were extracted and normalized using RMA (R/bioconductor package ‘oligo’). Probesets with log2-expression levels of >5 in at least three samples were subjected to differential expression analysis using limma and multiple-testing correction according to Benjamini and Hochberg (Benjamini and Hochberg, 1995) (R/bioconductor package ‘limma’). GO analysis was performed using the STRING database (version 10.0; http://string-db.org). False discovery rate (FDR) was calculated according to the method of Benjamini and Hochberg (Benjamini and Hochberg, 1995; Franceschini et al., 2013).

### Tissue preparation for fluorescent immunochemistry

For immunohistochemistry, mice were deeply anaesthetized with CO_2_ and immediately prepared for tissue preservation. Mice were transcardially perfused with 1% PBS (pH 7.4) followed by 4% PFA (pH 7; Roti^®^-Histofix, Germany) for 12 min (Gage et al., 2012; Kohler et al., 1999). Brains were carefully removed and post-fixed in 4% PFA (pH 7; Roti^®^-Histofix) for 12-72 h at 4°C, then dehydrated in 30% sucrose in ddH_2_O at 4°C for 24-48 h. Brains were cut into 30-µm coronal sections using a cryotome. Free-floating coronal brain sections were washed 3×10 min in 1% PBS (pH 7.4), blocked in blocking solution [1% (w/v) BSA, 0.5% (v/v) Triton X-100 in PBS] for 45 min at room temperature (RT; approx. 22°C) and incubated with primary antibody overnight at 4°C. Antibodies were diluted separately in blocking solution [polyclonal rabbit anti-Na_v_1.1, 1:200; rabbit anti-Na_v_1.2, 1:200; rabbit anti-Na_v_1.6, 1:200; rabbit anti-GABRA2, 1:500 (all Alomone Labs, Israel)] and co-stained with chicken anti-NeuN (1:500; Millipore, Germany) or mouse anti-MAP2 (1:1000; Sigma-Aldrich, Germany). After overnight incubation, sections were washed 3×10 min in 1% PBS (pH 7.4) and incubated with secondary antibodies in blocking solution for 2 h at RT. Sections were incubated with donkey anti-rabbit IgG Alexa Fluor 488 (1:500) and goat anti-chicken IgY Alexa Fluor 647 (1:500) or donkey anti-mouse IgG Alexa Fluor 647 (1:500; all Life Technologies, Germany). To counterstain nuclei, sections were incubated with DAPI (2 µg/ml; Thermo Fisher, Germany) for 5 min at RT and washed 3×10 min in 1% PBS (pH 7.4). After washing, the sections were mounted with Fluomount (Sigma-Aldrich). Confocal microscopy was performed with an inverted Leica SP8 microscope equipped with lasers for 405, 488, 552 and 638 nm excitation. Images were acquired with a 40×1.3 oil objective; image pixel size was 80 nm. The following fluorescence settings were used for detection: DAPI: 430-470 nm; AF488: 500-550; AF555: 560-600; AF647: 650-700. Images were scanned in a sequential fashion to avoid bleed-through. AF488, AF555 and AF647 were recorded with hybrid photo detectors (HyDs), and DAPI with a conventional photomultiplier tube. Overview images with high resolution were obtained by stitching.

### Acute slice preparation

Mice were deeply anaesthetized with CO_2_ before decapitation. Brains were quickly removed and submerged in ice-cold cutting solution consisting of (in mM) 135 N-methyl-D-glucamine, 1.5 KCl, 1.5 KH_2_PO_4_, 23 NaHCO_3_, 0.5 CaCl_2_, 3.5 MgCl_2_, 0.4 ascorbic acid and 25 D-glucose (pH at 28°C: 7.4; osmolarity: 310-330 mOsm) for 60 s. Coronal slices (slice thickness: 300 µm) were cut on a vibrating microtome (HM 650 V, Thermo Scientific Microm, Walldorf, Germany). Slices were collected and submerged in artificial cerebrospinal fluid (ASCF) containing (in mM): NaCl (125), KCl (3), NaH_2_PO_4_ (1.25), NaHCO_3_ (25), CaCl_2_ (2), MgCl_2_ (2) and D-glucose (25), and left to recover for 1 h at 28°C and for another 1 h at RT. Both solutions were continuously perfused with 95% O_2_/5% CO_2_ to maintain a pH of 7.4. For electrophysiological analysis, slices were transferred to a recording chamber mounted on the stage of a microscope (Zeiss Axioskop FS with a 40×, 0.75 NA objective). The recording chamber was continuously perfused with artificial cerebrospinal fluid (ACSF). The recording temperature was maintained at 30°C with the help of a temperature controller (Automatic Temperature Controller TC-324B, Warner Instrument Corp., CT, USA).

### Electrophysiological field recordings

The CA1 pyramidal cell layer was visualized and identified by means of an upright microscope equipped with differential interference contrast (DIC)-infrared optics. Infrared images were acquired with the help of a CCD camera and controller (Orca-ER, Hamamatsu, Shizouka, Japan). The electrodes for field recordings were fabricated from borosilicate glass capillaries (OD: 1.5 mm, ID: 0.86 mm; Hugo Sachs Elektronik-Harvard Apparatus, March-Hugstetten, Germany) and were filled with 1 mM NaCl solution. The electrodes were connected to the headstage of the amplifier (ELC; npi electronic, Tamm, Germany) via a chlorided silver wire. A silver/silver chloride pellet immersed into the recording solution served as reference electrode.

Electrode capacitance and resistance were compensated and bias and offset current were zeroed before the start of recordings. Evoked population spikes were recorded from CA1 pyramidal neurons after placing a monopolar or bipolar stimulation electrode in the SCC pathway. The stimulation intensity was increased in a stepwise fashion to obtain the optimal stimulation intensity (∼60% of the maximal response).

### Data acquisition and analysis

Recorded voltage signals were amplified (×20), filtered at 10 kHz and digitized at a sampling rate of 5 kHz. Data acquisition and generation of command pulses was accomplished by means of an analog-digital converter (CED Power1401; Cambridge Electronic Design, Cambridge, UK) in conjunction with the Signal data-acquisition software (version 6; Cambridge Electronic Design). Data analysis was performed using IGOR Pro 6 (WaveMetrics, Lake Oswego, OR, USA) together with the NeuroMatic IGOR plugin (www.neuromatic.thinkrandom.com).

### Image analysis

Images of coronal hippocampal slices were analyzed with Fiji 1.50 g (Schindelin et al., 2015). Regions of interest were selected and quantified as mean pixel intensity. To identify the StP (CA1-CA3), NeuN images were thresholded using the mean gray value autothreshold after median filtering of the image (radius 15). For DG, mean pixel intensity of cell bodies in the stratum granulare (StG) were measured. For GABRA2, signal was measured in CA1 (StP) for cell bodies and in StR CA1 for the dendritic field. The inverse mask of the pyramidal cell layer was used to quantify signal intensity in the dendritic compartment. All values were normalized to WT. Intensities were measured on the original, non-filtered images.

### qRT-PCR

Total mRNA was obtained from brain samples using TRIzol (Ambion) according to the manufacturer's protocol. DNA was depleted using the Mini RNeasy kit (Qiagen, Germany). cDNA was synthesized from purified mRNA by reverse transcription using Superscript III reverse transcriptase (Invitrogen) and random primers according to the manufacturer's manual. For qPCR cDNA amplification, Hot Start Taq (New England Biolabs, MA, USA) was used with SYBR Green for amplicon detection. All primers were used with an optimal efficiency rate of 2.0±0.5. Target gene signal was normalized to *Ppia* as reference gene using the comparative ΔΔC_T_ method (Schmittgen and Livak, 2008). Normalization to *18S* gave similar results. Runs were performed on a Lightcycler 96 (Roche Bioanalytics, Germany). Primers used in this study were (5′ to 3′): *Scn1a*, GAATCCCAAGCCAGACAAA and ACCATCTCTGGAGGAATGT; *Scn2a*, ACAGGAATTTATACTTTTGAATCA and AGTATCATGACGTCAGACAG; *Scn8a*, CTTCAGTGTCATCATGATGG and GCCCACGATTGTCTTCA; *Gabra2*, GAAAGGCTCCGTCATGATAC and GCTTGTTCTCTGGCTTCTT; *Gabbr2*, CTACGACGGTCTTACTCTCA and GGCCTCTCTCCTTTGTCTA; *Pum2*, AGCAACCAAGCACTAACC and CCAGGTCCATGAGAGAATAAAG; *Ppia*, GTCAACCCCACCGTGTTCTT and CTGCTGTCTTTGGAACTTTG; and *18S*, GAAACTGCGAATGGCTCATTAAA and CCACAGTTATCCAAGTAGGAGAGGA.

### Western blot

To analyze protein expression in Pum2 GT mice, brains were homogenized in RIPA buffer [150 mM NaCl, 1.0 vol% NP-40, 0.5% (w/v) sodium deoxycholate, 0.1% (w/v) SDS, 50 mM Tris-HCl pH 8.0, complete protease inhibitor (Roche)]. Proteins were transferred to a nitrocellulose membrane (pore size 0.2 µm). Membrane was blocked in blocking buffer [2% (w/v) BSA, 0.1% (v/v) Tween 20, 0.1% (w/v) sodium azide in 1× TBS pH 7.5] for 1 h. Pum2 was detected by incubation with polyclonal rabbit anti-Pum2 antibody (1:10,000; Abcam, Cambridge, UK) in blocking buffer. Protein bands for β-actin served as loading control and were detected with mouse anti-β-actin (anti-ACTB) antibody (1:2000; Sigma Aldrich). Proteins were visualized by incubation of the nitrocellulose membranes with secondary anti-rabbit antibody (1:10,000; Li-Cor, Germany) in blocking buffer. For quantification, the Pum2 signal was normalized to the loading control. Quantification of optical density (OD) was performed using Image Studio Lite Software (Li-Cor).

### Statistics

Data are presented as means±s.e.m. Statistics were calculated using the software GraphPad Prism (version 5; GraphPad, San Diego, CA, USA). Unpaired two-tailed Student's *t*-test was used to determine *P*-values. *P*<0.05 was considered statistically significant if not stated otherwise.

## Supplementary Material

Supplementary information

First Person interview
