# Supplementary material for: Pumilio2-deficient mice show a predisposition for epilepsy
Source: Dis Model Mech. 2017 Nov 1;10(11):1333–42. doi: 10.1242/dmm.029678 (PMC5719250; doi:10.1242/dmm.029678)
Supplement: Supplementary information [file dmm-10-029678-s1.pdf]

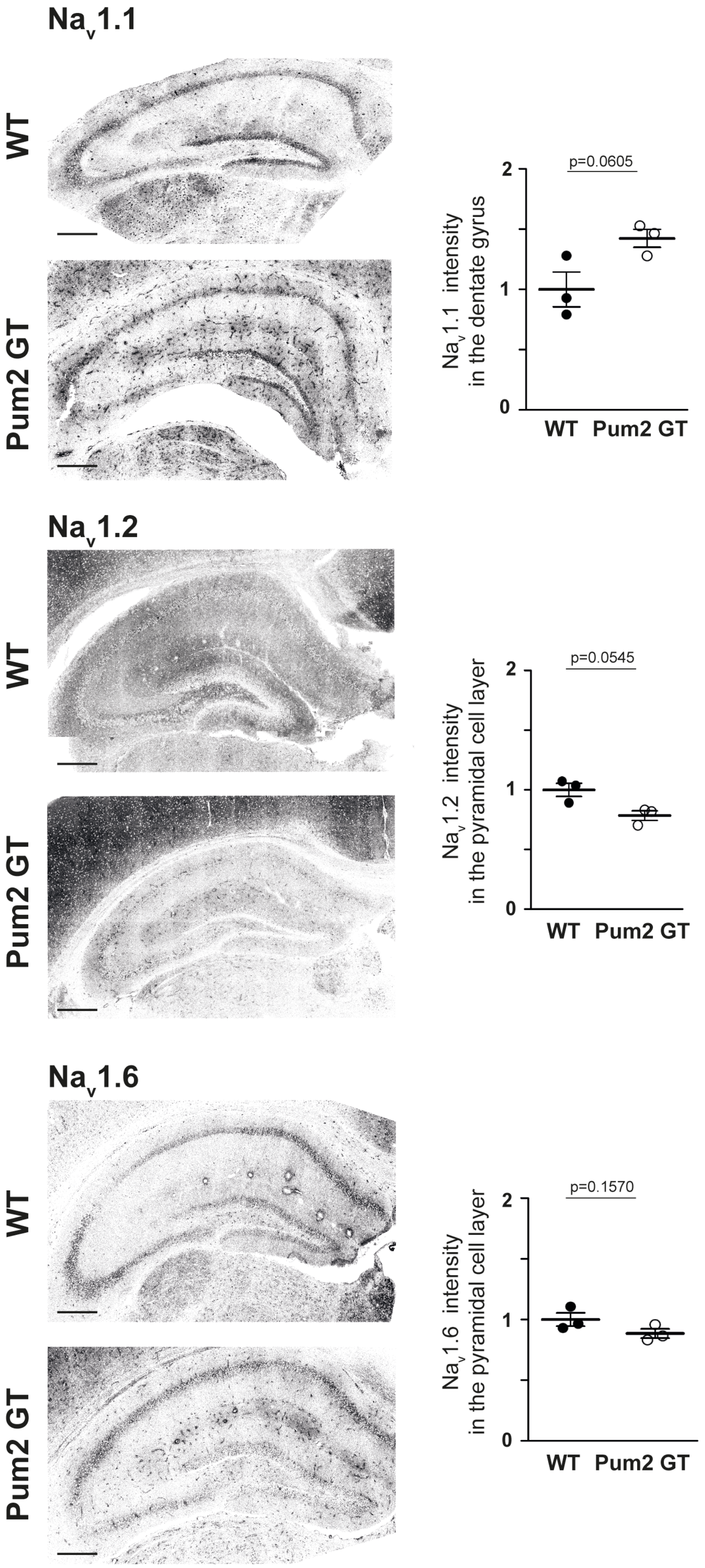

Disease Models & Mechanisms • Supplementary information

Fig. S1. (related to Fig. 4) Weaned Pum2 GT mice show no statistically significant alterations in hippocampal Na_v1.1, Na_v1.2, Na_v1.6 expression.

Representative immunohistological stainings for Na_v1.1, Na_v1.2, Na_v1.6 of weaned WT and Pum2 GT hippocampi. Quantifications are shown in the right panels (n=3 animals/group). Scale bars: 200 µm. Significance was determined using unpaired *t*-test.

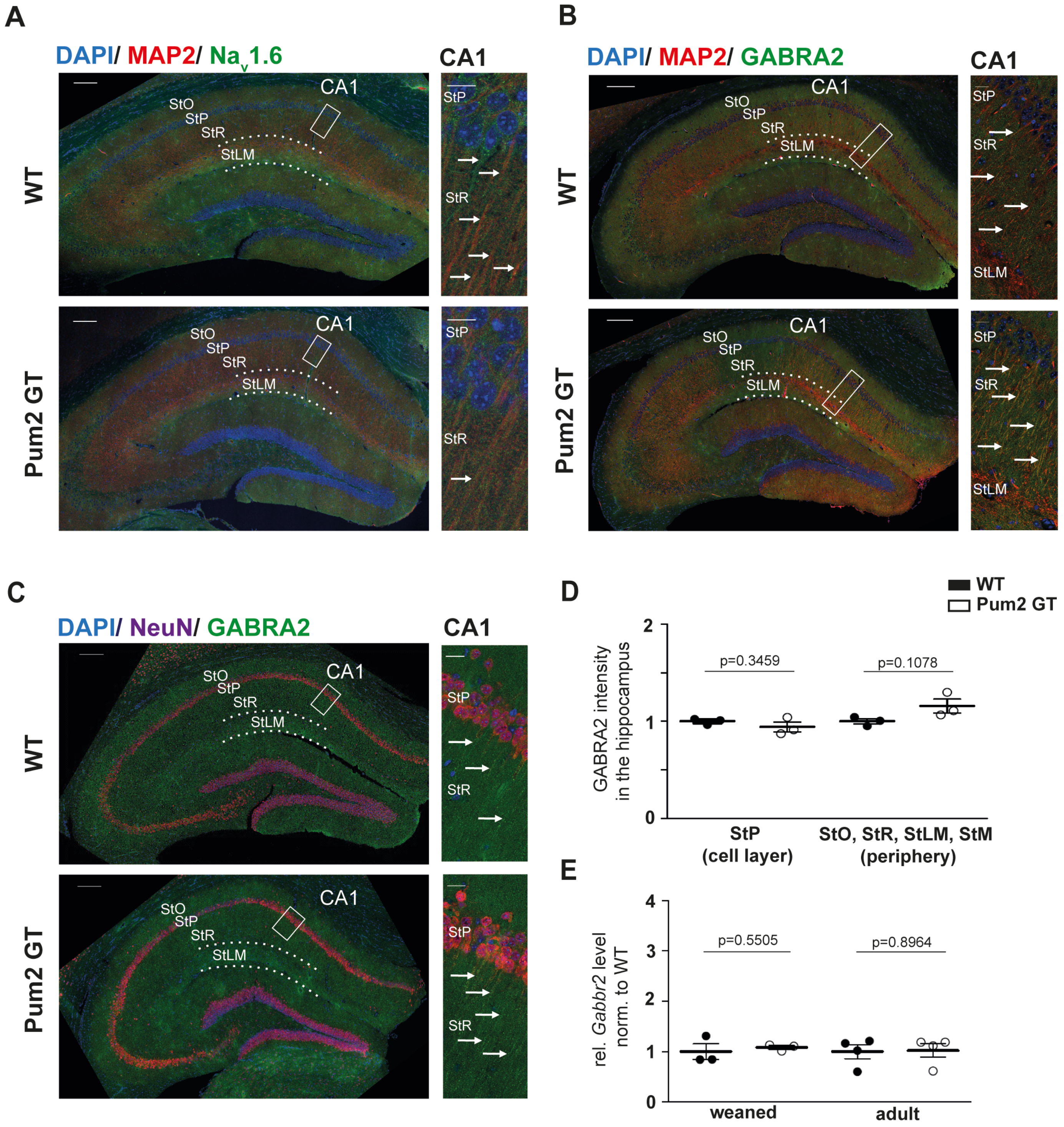

Disease Models & Mechanisms • Supplementary information

Fig. S2. (related to Fig. 4 and Fig. 5) *Gabbr2* level remains unaltered in weaned and 5 months old Pum2 GT brains.

(A) Representative co-staining of $Na_v1.6$ (green) and MAP2 (red) in WT and Pum2 GT hippocampi. DAPI was used to label the nuclei. Arrows denote dendritic colocalization of $Na_v1.6$ and MAP2. Scale bar: 200 µm, Inserts 20 µm.

(B) Representative co-staining of GABRA2 (green) and MAP2 (red) in WT and Pum2 GT hippocampi. DAPI was used to label the nuclei. Arrows denote dendritic colocalization of GABRA2 and MAP2. Scale bars: 200 µm, Inserts 20 µm.

(C) Representative co-staining of GABRA2 (green) and NeuN (red) in WT and Pum2 GT hippocampi. DAPI was used to label the nuclei. Arrows denote dendritic localization of GABRA2. Scale bars: 200 µm, Inserts 20 µm.

(D-E) Quantification of GABRA2 protein levels in the *stratum pyramidale* (CA1, CA3, DG) and periphery (*stratum oriens*; *stratum radiatum*, *stratum lacunosum-moleculare, stratum moleculare*) of the hippocampus (n=3 animals/group) (D) and *Gabbr2* mRNA levels (E) of WT and Pum2 GT mice (n≥3 animals/group). Significance was determined using unpaired *t*-test.

Table S1 (related to Fig. 2): Hand selected list of genes misregulated in Pum2 GT brains that regulate ion homeostasis. Significance level $p<0.05$.

Click here to Download Table S1

Disease Models & Mechanisms • Supplementary information
