# Supplementary material for: Pumilio2-deficient mice show a predisposition for epilepsy
Source: Dis Model Mech. 2017 Nov 1;10(11):1333–42. doi: 10.1242/dmm.029678 (PMC5719250; doi:10.1242/dmm.029678)
Supplement: First Person interview [file dmm-10-029678-s2.pdf]

© 2017. Published by The Company of Biologists Ltd | Disease Models & Mechanisms (2017): doi:10.1242/dmm.029678: Supplementary information

FIRST PERSON

First person – Philipp Follwaczny

First Person is a series of interviews with the first authors of a selection of papers published in Disease Models & Mechanisms, helping early-career researchers promote themselves alongside their papers. Philipp Follwaczny is first author on 'Pumilio2-deficient mice show a predisposition for epilepsy', published in DMM. Philipp is a medical student in his practical year under the supervision of Prof. Dr Michael Kiebler and Dr Bastian Popper at the Ludwig-Maximilians-Universität München, Germany, working in experimental epilepsy research.

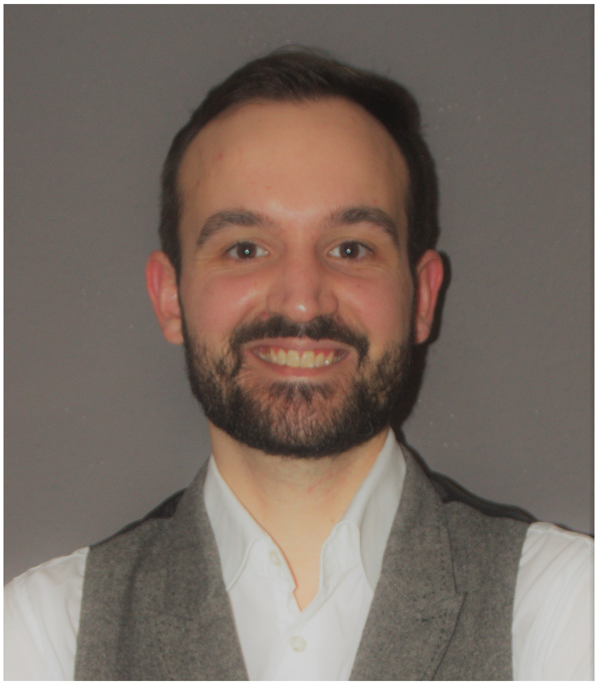

Philipp Follwaczny

How would you explain the main findings of your paper to non-scientific family and friends?

Epilepsy is a common neurological disease. While the cause of this disease is poorly understood, many neuronal proteins have been linked to the genesis of epilepsy, one of them being the RNA-binding protein Pumilio 2 (Pum2). The function of Pum2 is to control and direct RNA in the cell. RNA, the blueprint of DNA, is part of the protein production process, and Pum2 plays a role in making sure that functional proteins are correctly produced and strictly regulated. In neurons, dysregulation of proteins crucial to cell signaling could potentially cause overactive neuronal networks, leading to the development of epilepsy. In order to investigate the underlying mechanism of epilepsy, we had a look at some proteins that are crucial for cell signaling, e.g. ion channels and ion channel receptors such as voltage-gated sodium channels and GABA receptors in mice. Our investigations revealed that these proteins are dysregulated in mice lacking Pum2. This could lead to overactive neurons and potentially to the development of epilepsy.

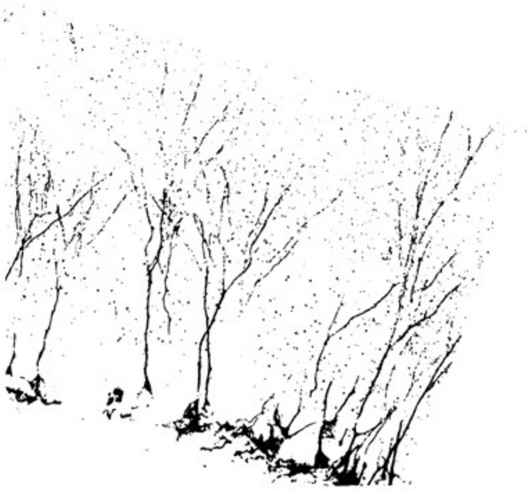

Young growing neurons in the hippocampus

Philipp Follwaczny's contact details: Department for Cell Biology, Biomedical Center, Medical Faculty, LMU, 82152 Planegg Martinsried, Germany.

E-mail: philipp.follwaczny@med.uni-muenchen.de

What are the potential implications of these results for your field of research?

We found that Pum2 is an important target in the development of epilepsy in mice, and particularly for congenital forms. The results indicate that Pum2 should be the subject of further study in relation to epilepsy, to improve our understanding of the disease and develop new treatment strategies.

What are the main advantages and drawbacks of the model system you have used as it relates to the disease you are investigating?

We used mice for our research, which have the advantage of a short generation time and a short lifespan. This allowed us to investigate the temporal development of seizures and the underlying molecular changes. Another advantage is the genetic matching of all replicates through inbreeding, providing a good level of confidence. Finally, we used a genetic approach, not an induced system, which works very well in mice. One of the biggest drawbacks in my opinion is the handling of mice. Sure, they are small, but they are still animals and we need a certain number of replicates. I think cell culture or flies might be easier to work with.

What has surprised you the most while conducting your research?

At the beginning, I was really focused on a few special targets and the whole project appeared to be clear. However, the longer it took, the more targets came up that were associated with Pum2 and epilepsy. I was quite surprised how big this number was and the amount of time

Disease Models & Mechanisms

it would take to have a look at all of them. As the research continued, the trend then reversed with the number of targets decreasing to around four really interesting proteins. This up and down during the time of my research was quite surprising for me.

"It's crucial to have motivational people around you, as science can be really frustrating at the beginning."

Describe what you think is the most significant challenge impacting your research at this time and how will this be addressed over the next 10 years?

I think we are still at the beginning of understanding how changes in the expression of Pum2 or other RNA-binding proteins might lead to epilepsy. In order to understand how molecular changes could cause an overactive neural network, I think it is really important to combine molecular work on the one hand with extensive functional work like electrophysiology on the other hand. Also, methods for imaging of the whole brain *in vivo* and *in vitro* will become more and more important to get a view of the genesis of epilepsy in the context of RNA-binding proteins. I think new experimental imaging technologies will address this well. In my opinion, the key point will be to transfer our mouse model to humans in order for it to be relevant for clinical neurology. This could be achieved, for example, through genomic studies on humans, epileptic surgery samples and also new imaging methods like SPECT or PET-CT. There is still a lot of work to be done.

What do you think is important at the start of a research career?

I had excellent supervisors who I could approach at any time for consultation. I think this is the most important thing right at the beginning of a scientific career. Another point is a clearly defined framework for a first project; there will be enough time afterwards for side projects. It's crucial to have motivational people around you, as science can be really frustrating at the beginning.

What's next for you?

First of all, I have to get my final admission as a physician and then my PhD. After that I would like to carry out some more research on epilepsy and finally get my postdoctoral qualification.

Reference

Follwaczny, P., Schieweck, R., Riedemann, T., Demleitner, A., Straub, T., Klemm, A. H., Bilban, M., Sutor, B., Popper, B. and Kiebler, M. A. (2017). Pumilio2-deficient mice show a predisposition for epilepsy. *Dis. Model. Mech.* **10**, doi:10.1242/dmm.029678.
